# WD-YOLO: A More Accurate YOLO for Defect Detection in Weld X-ray Images

**DOI:** 10.3390/s23218677

**Published:** 2023-10-24

**Authors:** Kailai Pan, Haiyang Hu, Pan Gu

**Affiliations:** School of Computer Science, Hangzhou Dianzi University, Hangzhou 310018, China; 211050008@hdu.edu.cn (K.P.); gupan@hdu.edu.cn (P.G.)

**Keywords:** YOLO, weld defects detection, attention mechanism

## Abstract

X-ray images are an important industrial non-destructive testing method. However, the contrast of some weld seam images is low, and the shapes and sizes of defects vary greatly, which makes it very difficult to detect defects in weld seams. In this paper, we propose a gray value curve enhancement (GCE) module and a model specifically designed for weld defect detection, namely WD-YOLO. The GCE module can improve image contrast to make detection easier. WD-YOLO adopts feature pyramid and path aggregation designs. In particular, we propose the NeXt backbone for extraction and fusion of image features. In the YOLO head, we added a dual attention mechanism to enable the model to better distinguish between foreground and background areas. Experimental results show that our model achieves a satisfactory balance between performance and accuracy. Our model achieved 92.6% mAP@0.5 with 98 frames per second.

## 1. Introduction

**Figure 1:**
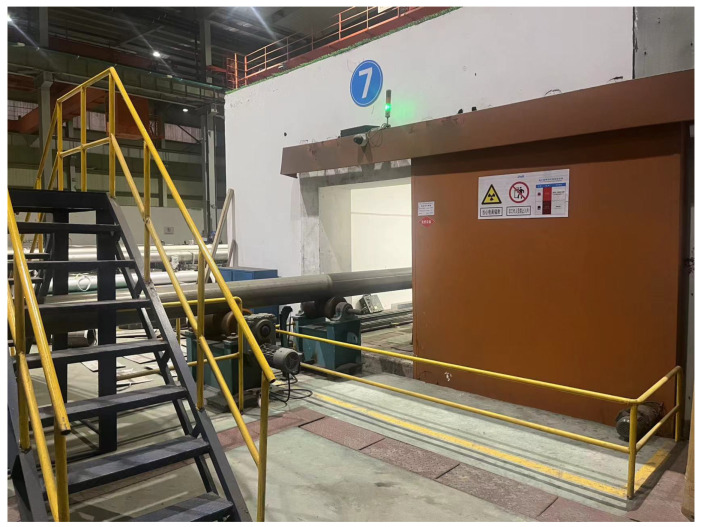
X-ray equipment. The transport device can automatically transport the steel pipes to the shooting room and automatically transport them out after the shooting is completed.

Defect detection in weld seams has always been one of the key research issues in the industry. Whether it is manual welding or robotic automatic welding, welding defects will inevitably occur. To solve this problem, people have adopted various methods to detect the quality of steel pipes. X-ray image defect detection is an important method of non-destructive testing (NDT) [1,2]. It is widely used in the quality inspection of steel pipe welds due to its low cost and high speed compared to other methods, such as computed tomography (CT) [3]. Figure 1 shows the equipment for taking X-ray images of steel pipe welds. Each steel pipe is sent into the shooting room by an electric wheel and sent out of the shooting room by an electric wheel after the shooting is completed. The process of taking X-ray images no longer requires manual work, but the evaluation of X-ray images still relies on manual labor [4].

This method is not only time-consuming and labor-intensive, but also subject to the subjective judgment and work experience of the radiologist, resulting in unreliable results. Long working hours can cause workers to lose concentration, resulting in some defects not being detected. Steel pipes used to transport oil or natural gas over long distances often have harsh working conditions, are difficult to maintain, and are always under high pressure. The weld is the weakest area of the steel pipe. If there are untreated defects in the weld, it may cause the steel pipe to leak. The leaked oil and natural gas will not only bring the risk of combustion and explosion, but also cause irreparable damage to the environment. Therefore, it is crucial to propose a weld defect detection model.

**Figure 2:**
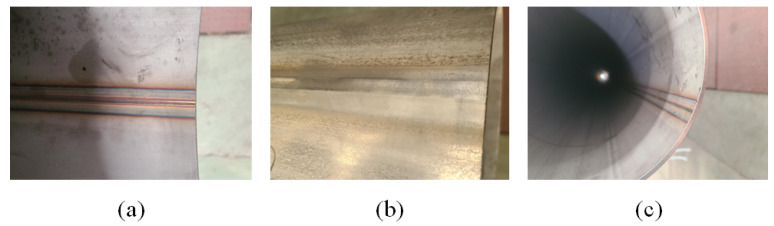
Images of the weld seam: (**a**) the inside of the weld; (**b**) the outside of the weld; (**c**) the cross-section of the steel pipe and the weld.

**Figure 3:**
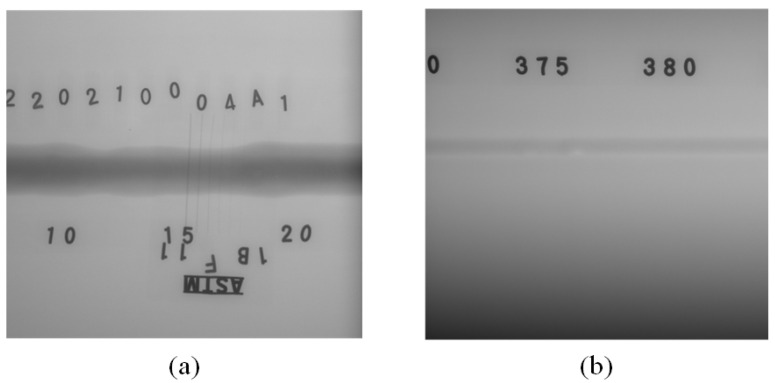
X-ray images of the weld seam: (**a**) high-contrast X-ray image; (**b**) low-contrast X-ray image.

There are already some weld defect detection models based on convolutional neural networks, but they often do not consider the image quality of the weld. In practical production, the contrast of the weld seam image can vary significantly. The weld seam in Figure 2a has a thicker seam due to a thinner steel pipe thickness, resulting in a clear boundary between the seam area and other background areas, which is easy to separate. The shape of the weld seam investigated in this paper is depicted in Figure 3. The exterior and interior of the steel pipe have been polished, and the weld seam has the same wall thickness as the steel pipe itself. This results in a low contrast X-ray image, and such low-contrast images can make defect detection extremely difficult.

Moreover, the background of the weld images is complex, there is no clear boundary between the defects and the background, and there are many types of welds with large size differences. The above factors bring great challenges to weld defect detection.

At present, many models based on convolutional neural networks have been applied to weld defect detection and have achieved certain results. However, most of these models only use advanced target detection models and do not consider the characteristics of weld defects. It often cannot achieve good results when detecting defects with blurred edges and unclear features.

In this paper, we propose a gray value curve enhancement (GCE) module to improve the image’s contrast. Second, we propose a high-precision target detection model called WD-YOLO for detecting defects in weld images. The backbone can preserve fine-grained information in weld images, extracting and fusing information in images more effectively. Finally, we propose a dual attention mechanism for the YOLO head. We use different attention modules for different defect sizes, which improves the model’s ability to segment the foreground from the background while accounting for the model’s computational requirements. We tested our object detection model on a self-built weld dataset and achieved good results. In summary, our contributions are as follows:

(1) A simple and novel image enhancement module named GCE is proposed. It can remove bad pixels in the image and can enhance the contrast of the image based on the gray value curve. Moreover, this module can be very easily applied to other weld defect detection models.
(2) We propose an efficient backbone that adopts a high-performance feature extraction module and many lightweight designs, making the model in this article have both high speed and high performance.
(3) We design a dual attention mechanism tailored to different types of defect characteristics and the actual needs of industrial production. Different attention modules are designed for different defect sizes to balance model accuracy and speed, and to significantly improve model recall rate.

The organization of this paper is as follows. Section 1 reviews related work on X-ray defect detection in weld seams. Section 2 introduces the process of the GCE module. Section 3 introduces the proposed NeXt backbone, dual attention, and WD-YOLO weld seam defect detection model. Section 4 presents comparative experiments as well as the ablation results of the model. Finally, Section 5 provides a summary and discussion of the paper.

## 2. Related Work

In the field of weld defect detection, there have been many traditional machine vision-based methods. Shao et al. [5] proposed a method based on low-threshold segmentation and Hough transform, which can detect weld defects with high certainty and avoid false positives caused by noise. Zou et al. [6] used Kalman filtering to detect the continuity of defect trajectories in image motion sequences, achieving real-time detection while avoiding false alarms and demonstrating good robustness. Sun et al. [7] proposed a method based on Gaussian mixture models to extract defect feature regions. They designed detection and classification algorithms based on region features and determined the classifier’s parameters through testing in actual production, achieving favorable results. Sun et al. [8] used fuzzy pattern recognition to automatically detect various defects based on the variance and contrast near the defects. Their detection method is simple and fast, enabling real-time detection. Malarvel et al. [9] presented a weld defect detection and classification method based on a multi-class support vector machine (MSVM), which can actively detect weld defects. Chen et al. [10] employed fast independent component reconstruction for images with defects and used a global threshold to segment porosity defects from difference images, enabling the detection of various porosity defects without manual labeling. Li et al. [11] used fast discrete curvelet transform and the maximum between-class variance method to denoise the image and extract the weld area, followed by fitting the grayscale curve with a third-order Fourier curve to segment the defect area. Dang et al. [12] proposed a method that calculates the peak–valley index and the defect index from the grayscale curve of the region of interest, determining the presence of defects through comparison.

With the advancement of deep learning technology, deep learning has been applied to solve various problems [13,14,15,16,17,18], and more researchers have been exploring the application of deep learning techniques to weld seam X-ray image defect detection. Jocher et al. [19] proposed the YOLOv5 model, which has been iterated to version 7.0 in 2022. Due to its excellent detection performance, YOLOv5 is currently used in many tasks [20]. YOLOv8 is the latest YOLO series model proposed by Ultralytics. YOLOv8 has higher model performance than YOLOv5. Chen et al. [21] proposed an AF-RCNN with attention mechanisms and combined ResNet with Feature Pyramid Network (FPN), achieving better accuracy and lower training loss compared to Faster-RCNN. Tao et al. [22] used an attention multi-hierarchical feature fusion network (AMHNet) to recognize defects and achieved excellent performance on the NPU-DRD dataset. Li et al. [23] introduced a novel Trident Net, utilizing dilated convolutions to significantly improve model performance without additional parameters and computational cost. Dong et al. [24] introduced an unsupervised local deep feature learning method, achieving results comparable to supervised learning by generating pseudo-labels through alternating feature clustering and training CNN using these labels. Kumar et al. [25] proposed a fully automated weld seam detection framework (W-VIF). Zhang et al. [26] presented a convolutional neural network-based defect detection method, capable of effectively identifying circular defects in weld seams, even in images with a wide range of grayscale values. Cheng et al. [27] introduced GhostBottleneck modules and attention mechanisms in YOLOv5, significantly reducing model parameters, making it suitable for embedded devices. Liu et al. [28] proposed an improved model based on YOLOv3, using the EFE module and RMF module to make the model lightweight while also improving performance. Yang et al. [29] proposed a YOLO-Xweld based on YOLOv3-tiny. This model added the SPP module to the backbone and reduced the number of detection heads, greatly lightening the model, allowing the model to be deployed on embedded devices.

The aforementioned works focus primarily on images with relatively high contrast and do not address the issue of low contrast weld seam images that are frequently encountered in actual production. In images with low contrast, the aforementioned methods cannot complete training and detection. YOLOv5-Ghost uses 1 × 1 convolution to generate half of the feature maps, which greatly speeds up inference. However, because half of the feature maps are not obtained by convolution from the upper feature map, the model accuracy will decrease. YOLO-Xweld reduces the number of detection heads of YOLOv3 from 3 to 2, improving the computing speed and enabling it to be deployed in embedded devices. However, due to the removal of the detection heads, the model’s large target detection performance is insufficient. The aforementioned model’s excessive emphasis on enhancing speed leads to a decline in its overall performance. YOLO-Xweld improves model speed by sacrificing detection capabilities for large targets. However, there are many large defects in weld images, and the focus of weld inspection is the performance of the model rather than the inspection speed of the model. To address these concerns, we propose an effective weld seam image enhancement algorithm for low-contrast images, stabilizing the quality of images input into the YOLO model. Furthermore, we improved the YOLOv5 model by replacing the backbone with a NeXt backbone specifically designed for weld seam defect detection tasks and by adding dual attention mechanisms in the head. Experimental results demonstrate that our model outperforms mainstream detection models and existing weld seam defect detection methods, showing promising advantages.

## 3. Adaptive Enhancement

**Figure 4:**
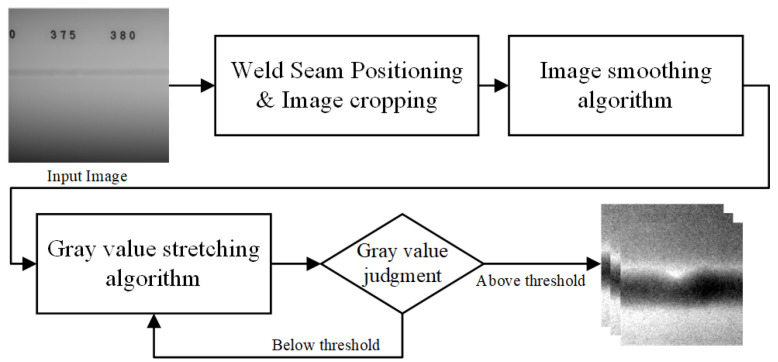
Adaptive contrast enhancement algorithm.

Due to the low contrast of X-ray images and the presence of image noise, some images cannot be directly used for training and detection. To the best of our knowledge, no one has proposed a solution to this problem. Therefore, we propose a gray value curve enhancement (GCE) module for image enhancement and image noise removal. Noise can impact the overall gray values of an image, resulting in ineffective results when gray value stretching algorithms are applied. The noise in the image is mainly divided into salt and pepper noise and Gaussian noise, but the number of noise points is very small. Therefore, supplying the entire image to methods such as median filtering or Gaussian filtering may result in the loss of edge information, leading to blurred and indistinguishable flaws. We propose a gray value curve improvement (GCE) module to improve the X-ray images. The processing workflow is illustrated in Figure 4.

**Figure 5:**
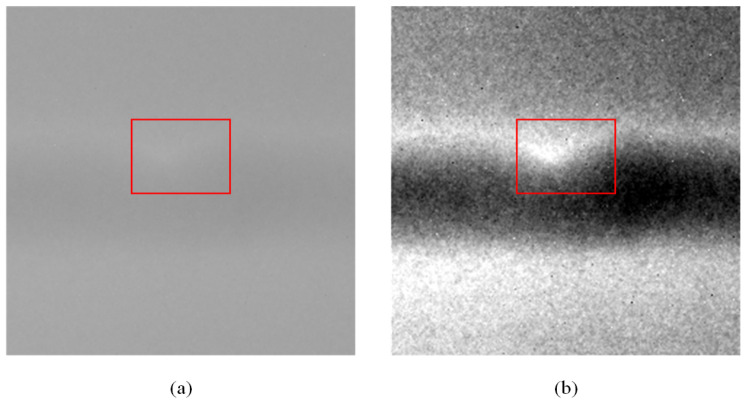
Images before and after enhancement: (**a**) original image, cropped from Figure 2b; (**b**) enhanced image. The red box in the image is the position of the defect.

Firstly, due to the highly automated process of capturing weld seam images, the position of the weld seam fluctuates only within a small range. Therefore, we can directly determine the position of the weld seam and crop it into small patches of 320 × 320 pixels (as shown in Figure 5a). Next, we analyze the histogram of the image, as it effectively reflects the distribution of pixels in the image. We use the histogram to find the location of the suspicious noise point and determine whether it is a noise point through its 8-field information. We set the value of the noise point to the average of the point values of its eight neighbors to remove the noise point. After removing bad pixels, the grayscale values of the image pixel values will be concentrated in a smaller range, and we remap this part of the pixel values to the range of 0–255. To reflect the differences between points with the same pixel value in different areas, we adopt a pixel value calculation method that combines four neighborhoods. The formula for remapping is as follows:

(1) $$P^{\prime }=255\times \frac{0.7P+0.3Q}{MAX-MIN},$$

where $P^{\prime }$ is the final mapping result, *P* is the original pixel value of the point, *Q* is the average of the four neighborhood pixels, MAX is the maximum pixel value in the original image, and MIN is the minimum pixel value in the original image.

This mapping method distinguishes points with the same pixel value, and the pixel values can be distributed as disparately as possible over a larger range, greatly enriching the information of the image.

(2) $$C=average(\sum_{\delta }\delta {\left(i,j\right)}^{2})$$

where δ(i,j)=|i−j| is value interpolation between adjacent pixel values, and the term average is for averaging.

If the *C* of the image is above the threshold, it indicates that the preprocessing is complete, and we output the image for training and detection. If the *C* of the image is below the set threshold, the above operations are repeated. Figure 5a is the image before enhancement, with a *C* value of 19.9. We set the threshold to 70, and the enhanced image is shown in Figure 5b. By comparing Figure 5a,b, we can easily observe that the image’s contrast is greatly enhanced with the GCE module. Usually, image enhancement can be completed in one iteration. For a small number of images, image enhancement can be completed in two iterations. The defects in the red frame have clear areas of high brightness, and this clear feature will significantly lessen the challenge of further detection.

**Figure 6:**
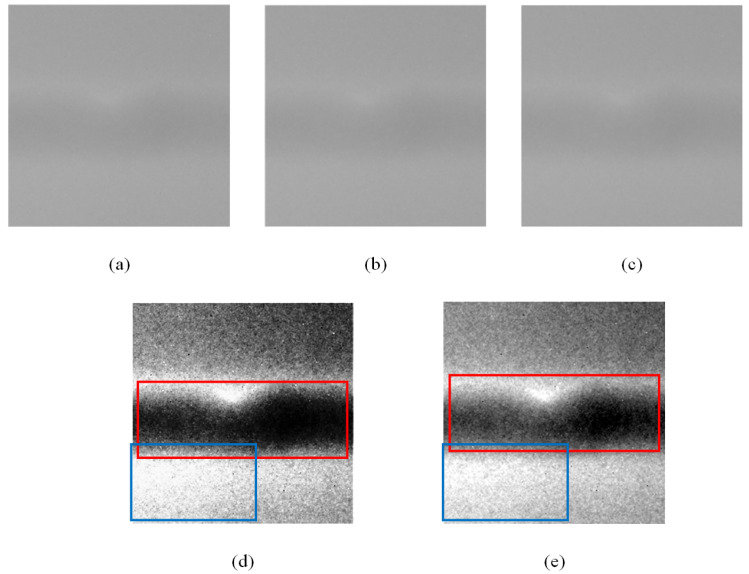
Images processed by different methods: (**a**) the original image; (**b**) image after Gaussian filtering; (**c**) image after median filtering; (**d**) image after histogram equalization; (**e**) image enhanced by GCE module.

The comparison diagram between the GCE module and other image processing methods is shown in Figure 6, in which all input images are processed by an image smoothing algorithm. It can be seen from the figure that the processing results of Gaussian filtering and median filtering have almost no effect on the weld seam image. The histogram equalization achieves effects similar to the GCE module. However, the image after histogram equalization will lose much of the gray value information in the image. Comparing the red boxes in Figure 6d,e, the image processed by the GCE module retains the light and dark relationship inside the weld, while the center of the weld in the image after histogram equalization has become completely black. Comparing the blue boxes of the two pictures, we can find that there are more differences in the pixel values of the images processed by the GCE module. Finally, histogram equalization can also cause bad pixels to reappear in the image. In summary, the GCE module we proposed can greatly enhance the contrast of the image while retaining the greatest extent of image information.

To ensure the fairness of the experiment, all experiments use enhanced images for training and testing, except for the comparison experiment of the GCE module.

## 4. Proposed Method

### 4.1. WD-YOLO

**Figure 7:**
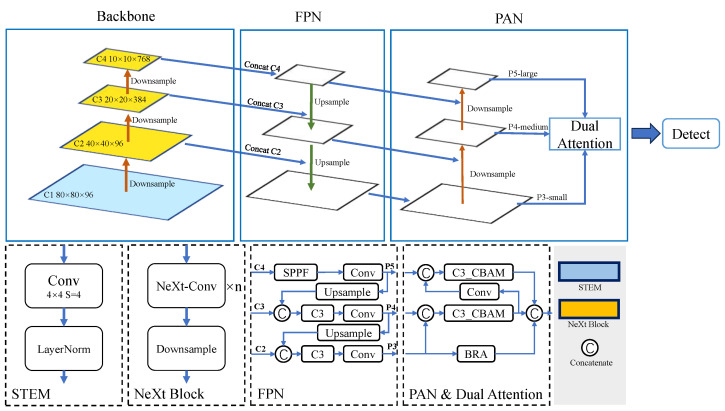
Structure of WD-YOLO. WD-YOLO adopts a new backbone and embeds a dual attention mechanism in the head while retaining the feature pyramid and path aggregation network structure.

We propose the WD-YOLO model for weld defect detection, and the structure of WD-YOLO is shown in Figure 7. The original weld image is processed by the stem layer for down-sampling and initial feature extraction. Then we use three stacked NeXt blocks to extract and fuse feature information in the feature map. Each NeXt block will halve the size of the feature map, and the final feature map output from the NeXt backbone is 1/32 of the original input weld image. In the YOLO head, we retained the feature pyramid network (FPN) and path aggregation network (PAN) structure from YOLOv5. This structure can combine feature maps of different sizes, enabling the model to detect targets of different scales. Additionally, we added dual attention methods for various levels of the feature pyramid. We created the C3_CBAM layer by adding the CBAM attention mechanism [30] to the C3 layer to detect larger objects. Further, we added the BRA attention module [31] to the lower-level layers to improve the model’s ability to effectively recognize small objects.

### 4.2. Stem Layer

The stem layer is used to remove redundant information in the original image, reduce the resolution of the image, and extract the initial features of the image. YOLOv5 down-samples the original image using a 6 × 6 convolution with a stride of 2. However, such a large convolution kernel will result in the loss of detailed information, which is crucial for the detection of weld defects. In WD-YOLO, inspired by the patchify stem in Vit [32], we propose the stem layer, which consists of non-overlapping convolution kernels of small convolution kernels and layer normalization (LN) layers. The structure of the stem layer is shown in Figure 6.

The non-overlapping convolutional layer is a convolution operation in which the stride and convolution kernel size are identical. Because some defects have the characteristics of small size and blurred boundaries, using large kernel convolution in the first layer of the input will lose a lot of important fine-grained information. Therefore, in the stem layer, we use 2 × 2 non-overlapping convolutions with a stride of 2, and the output feature map size is the same as a 6 × 6 convolution with a stride of 2.

Batch normalization (BN) is a prevalent strategy for training convolutional neural networks that can effectively address the gradient problem and accelerate the model’s convergence. However, an improper batch size configuration will diminish the performance of the model. Therefore, in WD-YOLO, we replace the BN layer with the LN layer. The LN layer normalize all features of each sample, which eliminates the impact of the batch size on the model performance and is more friendly to the training equipment.

### 4.3. NeXt Block

In this paper, we propose the NeXt block for feature extraction and fusion. We integrate some design concepts from Transformer in the NeXt block to enhance the performance of the model. The NeXt block consists of several NeXt-Conv layers and one down-sample layer.

**Figure 8:**
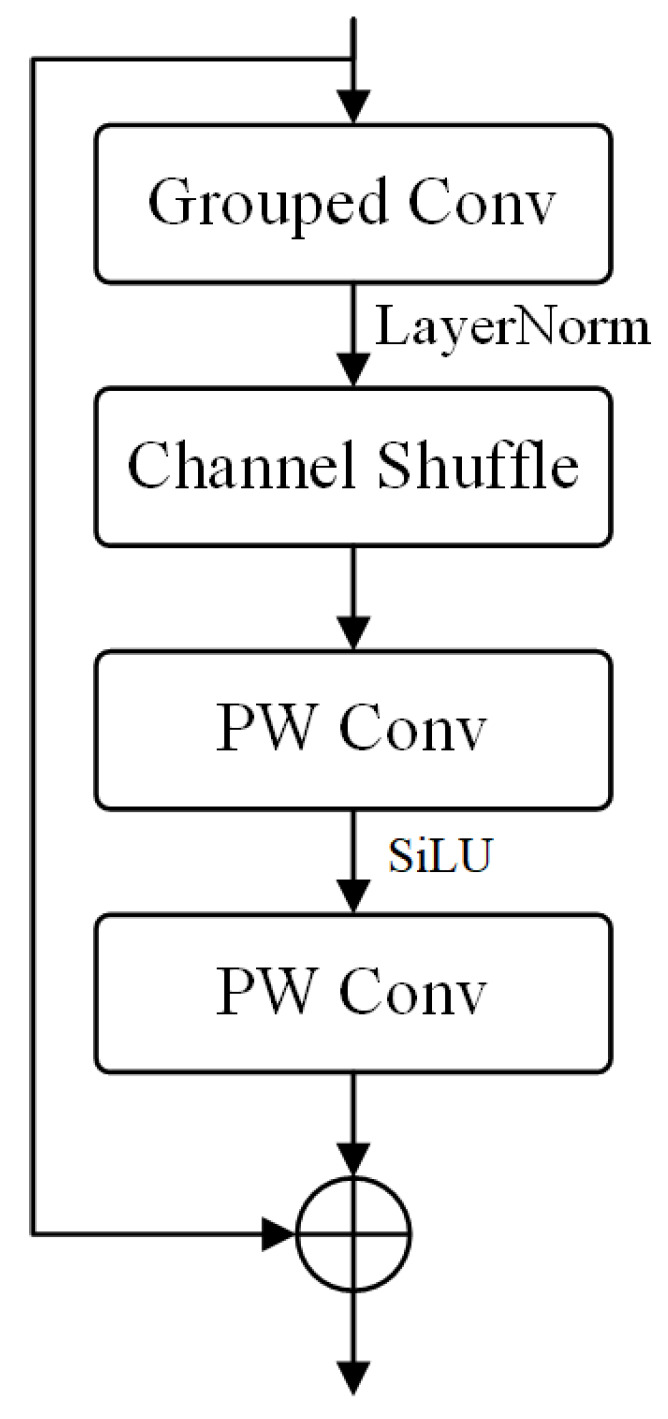
Structure of the NeXt-Conv layer.

The NeXt-Conv layer aims to increase the network’s ability to extract features by increasing its depth. As illustrated in Figure 8, the NeXt-Conv layer is composed of a layer of group convolution, a channel shuffle layer, and two pointwise (PW) convolution layers. Group convolution groups the input feature maps, convolves each group with a distinct convolution kernel, and then merges the resulting feature maps. This operation can considerably reduce the number of parameters and accelerate the model’s inference speed. We refer to the design of ConvNeXt [33], using a 7 × 7 convolution.

**Figure 9:**
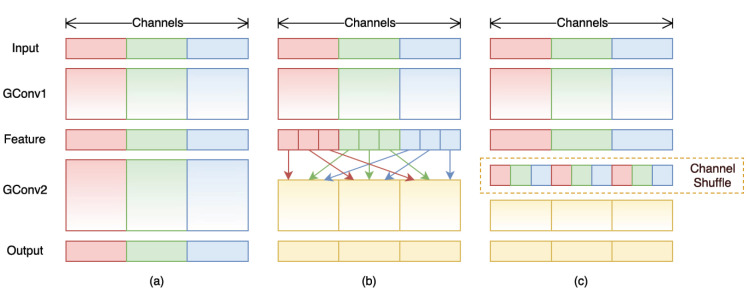
Channel shuffle with two stacked group convolutions: (**a**) two stacked convolution layers with the same number of groups. Each output channel only relates to the input channel within the group; (**b**) input and output channels are fully related when GConv2 takes data from different groups after GConv1; (**c**) an equivalent implementation to (**b**) using channel shuffle.

Due to the grouped convolution dividing feature maps into several small groups, all computations are performed within each group, resulting in a decrease in the feature correlation between different groups and ultimately leading to a drop in model accuracy. Therefore, we employed a combination of channel shuffle and PW convolution to address this issue. Channel shuffle, initially introduced in ShuffleNet [34], is used to increase channel interaction between different groups in grouped convolutions, and the process is illustrated in Figure 9. This operation recombines several sets of feature maps obtained from GConv1 into new groups. Specifically, it first rearranges G groups of N-dimensional feature maps into a G × N 2-dimensional matrix and then transposes the matrix. Subsequently, the transposed matrix is reconverted back into G groups of N-dimensional feature maps. Channel shuffling requires only a small amount of calculation to eliminate the barriers between different convolution groups, which significantly improves the expressive ability of the model.

Then, we utilize two pointwise (PW) convolutions to extract deep features. The channel design of these two PW convolution layers differs from the traditional bottleneck layer, with inspiration drawn from the concept of channel expansion first introduced in MobileNetV2. The first PW convolution layer is referred to as the expansion layer, where the channel expansion factor is determined by the expansion factor, set to four in our model. After passing through the first PW convolution layer, the number of channels becomes four times the original. The second PW convolution layer is called the extraction layer, aiming to extract features from the feature maps.

In traditional bottleneck layers, feature maps are mapped to a lower dimension and then restored to the original dimension. During this dimension transformation process, some loss inevitably occurs due to the inability of the low-dimensional features to fully represent the high-dimensional features, thus affecting the model’s accuracy. The NeXt layer transforms the input feature map into a higher dimension. The higher dimension makes the feature information richer and has more parameters for learning.

The down-sampling layer compresses the extracted features and expands the model’s receptive field. YOLOv5 uses a 3 × 3 convolution with a stride of 2 to extract the information of the input feature map and halve the size of the feature map. Since the input here is the feature map extracted by the network, which contains much less redundant information compared to the original image, we use the non-overlapping convolution for this operation. The structure of the down-sampling layer is like the stem layer, comprising an LN layer and a 2 × 2 convolution with a stride of 2.

The type and number of activation functions significantly impact the model’s performance. Activation functions often have very small gradients in the negative region, leading to the death of some neurons and information loss. Therefore, having fewer activation functions helps the model retain critical features. We added only the SiLU activation function in between the two PW convolution layers.

### 4.4. Dual Attention Mechanisms

The attention mechanism has been widely used in deep learning. In the task of X-ray weld defect detection, employing attention mechanisms can help the model better distinguish foreground and background [35], thus improving detection accuracy. This paper proposes a dual attention mechanism. Specifically, we design different attention mechanisms for different levels to improve the performance of the model while saving computation.

**Figure 10:**
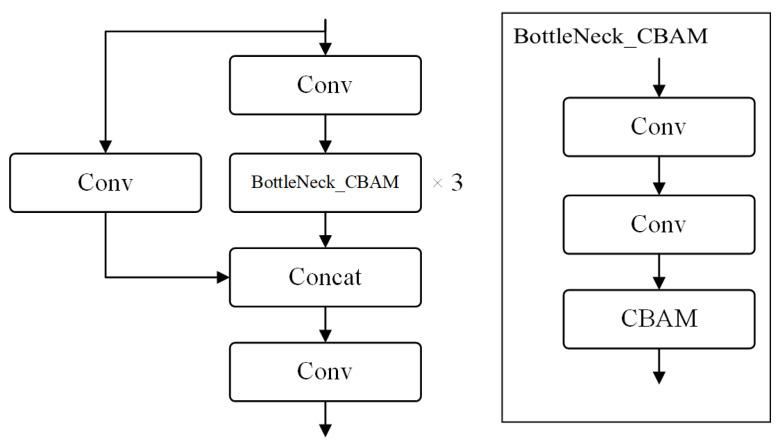
Structure of C3_CBAM and bottleneck_CBAM.

**Figure 11:**
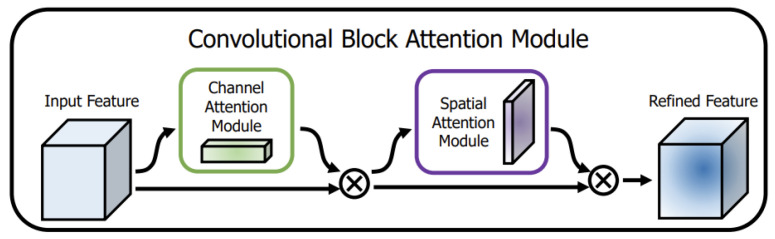
The overview of CBAM.

We incorporate the Convolutional Block Attention Module (CBAM) attention mechanism [30] into the C3 module in the large and the mid object detection layer, namely, C3_CBAM (Figure 10). CBAM consists of two independent sub-modules, namely the Channel Attention Module (CAM) and the Spatial Attention Module (SAM), as illustrated in Figure 11.

**Figure 12:**
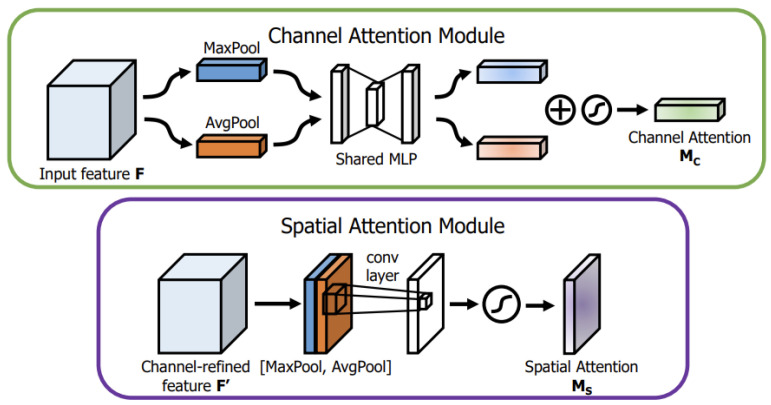
Diagram of each attention sub-module.

The CAM (Figure 12) first applies max-pooling and average-pooling operations to the input feature map F, which helps remove redundant information and compress the features. The pooled results are then fed into a shared multi-layer perceptron (MLP) for further compression. The compressed feature maps are added together and passed through a sigmoid activation function to obtain the final channel attention weight Mc. Finally, the channel attention weight Mc is multiplied with the input feature map F to obtain the output feature map F’ (channel-refined feature F’).

The SAM (Figure 12) focuses on the positional information in the image and takes the output feature map F’ from the channel attention as its input. The SAM first performs max-pooling and average-pooling operations along the channel dimension of F’ and then combines the pooled feature maps. The combined feature map is then compressed using a convolutional layer with a kernel size of 7 × 7. The compressed single-channel feature map is passed through a sigmoid activation function to generate the spatial attention weight Ms. Finally, Ms is multiplied with F’ to obtain the final output feature map with spatial attention F”.

In a model without an attention mechanism, the weight of all feature maps and each feature vector obtained by convolution is the same. These include defect features we need to detect and irrelevant background features. The CBAM attention mechanism can learn to give different weights to different feature maps and feature vectors, which can help the model better distinguish between the foreground and the background.

We utilize the Bi-Level Routing Attention (BRA) mechanism [31] in the small object detection layer to enhance the model’s ability to detect minute defects. BRA divides the self-attention calculation into two stages. In the first stage, BRA divides the input feature map into large patches and calculates the relationship matrix between large patches through self-attention. In the second stage, BRA will subdivide the large patch into smaller patches and only calculate the attention between related patches according to the relationship matrix calculated in the first stage. BRA is a lightweight self-attention mechanism based on sparse sampling, which has good results in small target detection.

## 5. Experiments

### 5.1. Experiment Environment

This experiment was conducted using the Windows 11 operating system, with an AMD Ryzen 9 5900X CPU and an NVIDIA RTX4090 GPU. The deep learning framework used is PyTorch 2.0.1, and the CUDA version is 12.1. The learning rate is 0.001, and the momentum factor is 0.937. The input image size is 320 × 320, and the batch size is 16. The model was trained for 300 epochs. Our model was trained for a total of 42 h.

### 5.2. Dataset

This experiment used a self-built dataset of welding seam defects, and the X-ray images were sourced from the small welding pipe workshop of Zhejiang Jiuli Hi-Tech Metals Co., Ltd., Huzhou, China. The dataset contains a total of 3153 weld defect images, including 724 pit images, 421 plate-hole images, 874 plate-injury images, 557 misalignment images, 217 inclusion images, 357 porosity images, and 363 undercut images. The images were divided into training and testing sets in an 8:2 ratio. Except for experiments that demonstrate the effectiveness of the GCE module, all images input to the model use the GCE module for adaptive contrast enhancement.

### 5.3. Evaluation Indicators

We use precision, recall mAP@0.5, and FPS as metrics. The precision indicates the proportion of real defects among all detected defects; higher precision corresponds to a lower false detection rate. It is calculated as shown in Equation (3):

(3) $$precision=\frac{TP}{TP+FP}.$$

The recall rate indicates the proportion of detected defects to all defects; higher recall corresponds to a lower miss detection rate. It is calculated as shown in Equation (4)

(4) $$precision=\frac{TP}{TP+FN}.$$

The mAP@0.5 indicates the average precision rate of all detection results with an intersection ratio greater than 0.5; mAP@0.5 is an important indicator to measure the overall performance of the model. We use GFLOPs and Params to measure the computational complexity of the model. Then, we use FPS to measure the speed of the model during inference. The higher the FPS, the faster the model.

Miss detection will treat unqualified steel pipes as qualified steel pipes. Unqualified steel pipes are at risk of rupture. Whether a pipe rupture occurs in a factory or in a residential building, it can cause huge economic losses and safety risks. False detection will cause the steel pipes to enter the repair assembly line, and the losses caused by incorrect inspections are much smaller than missed inspections. Therefore, the recall of the model is more important than the precision of the model.

### 5.4. Comparative Experiment

We compared our model with YOLOv8, YOLOv5, YOLOv5-Ghost [27], LF-YOLO [28], and YOLO-Xweld [29] on the self-built dataset to prove the superiority of WD-YOLO. YOLOv8 is the latest model of the current YOLO series. YOLOv5 is currently a model that is widely used in actual production. Yang [36] and others applied YOLOv5 to detect weld defects. YOLOv5-Ghost is a lightweight model of YOLOv5. LF-YOLO and YOLO-Xweld are two improved models based on YOLOv3.

The experimental results are presented in Table 1.

YOLOv8 has the highest accuracy rate, which is 2.3% higher than WD-YOLO, but the recall rate of YOLOv8 is 14.8% lower than that of WD-YOLO, and the inference speed of YOLOv8 is also slower than our model. The accuracy of YOLOv5 is lower than YOLOv8, but the recall rate is higher than YOLOv8; however, all indicators are lower than WD-YOLO. LF-YOLO also showed excellent performance on our self-built dataset. The recall rate of LF-YOLO reached 0.904, which is only 1.9% lower than WD-YOLO. The mAP@0.5 of LF-YOLO is only 1.2% lower than WD-YOLO. Due to the lightweight design of LF-YOLO, the inference speed of LF-YOLO is much faster than that of WD-YOLO, reaching 178 FPS. YOLO-Xweld is mainly used to detect small targets and deletes the detection head for detecting large targets. It performs poorly on our dataset, with a precision 7.1% lower than our model and a recall rate 12.3% lower than our model. Additionally, mAP@0.5 is 4.3% lower than our model. However, due to its lightweight design, the model’s inference speed is also much faster than our model.

In terms of model inference speed, our model is inferior to various lightweight models. However, because our model also adopts a lightweight design, the overall inference speed is better than the original YOLOv5 and YOLOv8 models. The primary limiting factor in the efficiency of weld quality inspection during actual production is predominantly attributed to the speed at which X-ray pictures are captured. Therefore, the inference speed of our model can still meet the requirements.

**Figure 13:**
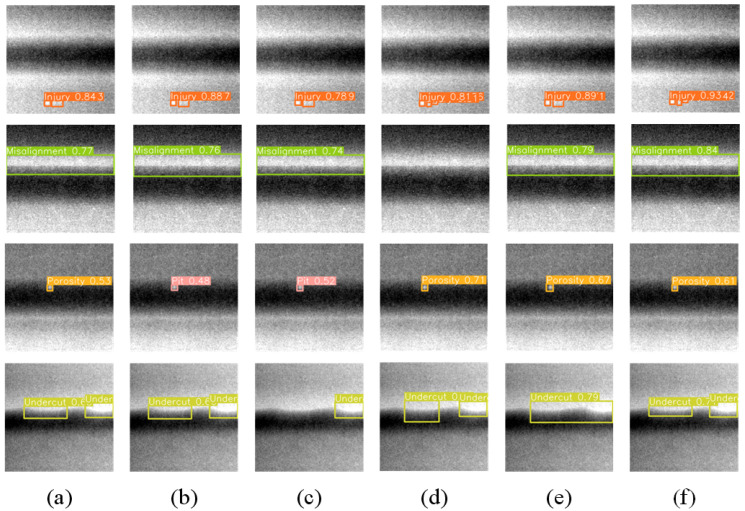
Detection results: (**a**) results of YOLOv5; (**b**) results of YOLOv8; (**c**) results of YOLOv5-Ghost; (**d**) results of YOLO-Xweld; (**e**) results of LF-YOLO; (**f**) results of WD-YOLO.

Figure 13 presents representative experimental results of the YOLO series models and other advanced weld defect detection models.

The characteristic of injury is that it often appears densely. As can be seen from Figure 13, only YOLO-Xweld and WD-YOLO detected three defects. Misalignment is a large-sized defect that usually spans several X-ray images. YOLO-Xweld does not detect this defect because it deletes the detection head of the large target. The shapes of porosity and pit are very similar, and the original YOLO series models both experienced false detections. In the detection of undercut defects, YOLOv5-L missed one defect, and LF-YOLO merged the two defects into one.

In actual production, the detection of defects does not mean that the entire steel pipe will be discarded. Workers will assign defective steel pipes to different maintenance lines based on the test results. Missing detection of defects will cause the steel pipe to be regarded as a qualified steel pipe, causing hidden dangers for future use. Incorrectly detecting defects will cause steel pipes to be assigned to the wrong maintenance lines, wasting time and human resources. Most of the defects detected by X-ray images are not visible on the surface, and workers need to repair the steel pipe based on the inspection results of the model. If the location of the detected defects is inaccurate or if multiple defects are merged into one defect, it will mislead workers and lead to incomplete repairs, causing safety hazards.

Based on the above analysis, we can conclude that WD-YOLO outperforms other state-of-the art models on our dataset.

### 5.5. Ablation Experiment

To validate the effectiveness of the proposed improvements, comparative ablation experiments were conducted using YOLOv5-L as the baseline model. Specifically, the NeXt backbone and the dual attention mechanism were separately tested as validation modules. The training outcomes are presented in Table 2.

It can be observed that directly applying the dual attention mechanism of this paper on YOLOv5-L has increased the recall rate by 6.3%; the accuracy rate has decreased by 1.7%; the mAP0.5 has increased by 3.1%; and the overall performance of the model has increased slightly. Using only the BRA attention mechanism will greatly reduce the accuracy of the model, indicating that BRA is not suitable for use alone in the task of weld detection. Using only the CBAM attention mechanism will improve the accuracy of the model, reaching 90.1%, which is also the structure with the highest accuracy among various improved models. However, the recall rate of the WD-YOLO network using only CBAM is low, and the model’s mAP@0.5 is also lower than WD-YOLO without an attention mechanism. According to the above experimental results, we can conclude that the single attention mechanism does not improve the model significantly, and the performance of the model even declines; however, the dual attention module can effectively improve the performance of the model.

Compared with the YOLOv5 with a double attention mechanism, the model proposed in this paper has a 3.9% increase in accuracy, a 5.1% increase in recall rate, and a 4.1% increase in mAP@0.5, indicating that the NeXt backbone is more suitable for weld detection tasks.

### 5.6. Batch Norm and Layer Norm Experiment

We also conducted comparative experiments on the improvement proposed earlier, which involves replacing BN with LN. As discussed earlier, BN normalization is performed per batch, thus being influenced by batch size, whereas LN normalization is performed across all features of each individual sample. This means that the model’s performance will not be affected by batch size, allowing for the use of smaller batches during training. Smaller batches, in turn, require less memory and GPU memory, making them more resource-friendly for training devices.

In the comparative experiments, we applied WD-YOLO with both BN and LN in the backbone, using different batch sizes for comparison. The experimental results are shown in Table 3. It can be observed that the WD-YOLO model with LN shows only a 1.6% difference in mAP@0.5 across various batch sizes, indicating relatively consistent performance. The slight performance decrease could be due to the continued use of BN in the C3 module and convolutional blocks in the head section. On the other hand, the model with BN in the backbone is more significantly affected by batch size, and it is apparent that, as the batch size increases, the model’s performance gradually improves.

### 5.7. GCE Module Experiment

We use a comparative experiment to prove the effectiveness of the GCE module. In this experiment, we use the image before enhancement and the image after enhancement, respectively.

Table 4 shows the experimental results of the GCE module experiment. Whether it is YOLOv5 or WD-YOLO, if the GCE module is not added to enhance the input image, it is almost impossible to detect defects normally.

## 6. Conclusions

This paper presents an enhancement algorithm for low-contrast X-ray images and an improved YOLOv5 network for weld defect detection. We adaptively remove image artifacts based on pixel distribution and image resolution, followed by an adaptive grayscale stretching based on the grayscale curve of the artifact-removed image to achieve image enhancement. WD-YOLO is our proposed enhanced network, where we design a new backbone for weld defect detection tasks. This backbone employs group convolutions, channel shuffling, and inverted bottleneck layers. Additionally, in the head part of WD-YOLO, we introduce an attention mechanism to adapt the model to small objects and complex imaging environments. Compared to models of similar scale like YOLOv5-L, our model exhibits superior detection performance and faster inference speed. While our detection model is slightly slower than lightweight models in terms of detection speed, its accuracy surpasses that of lightweight models. We deployed the model to analyze the weld quality inspection line of Jiuli Co., Ltd. In actual tests, the detection speed of our model is faster than the speed of taking X-ray images, which fully meets the speed requirements in actual production. However, Jiuli Co., Ltd. employees told us that there are still some very rare defects that are not included in our dataset, so our model does not have the ability to detect such defects. In future work, we will continue to improve our dataset so that our model can detect more types of defects.

## Figures and Tables

**Table 1:** Comparison with defect detection methods.

Method	Precision	Recall	F1	mAP@0.5	FPS	GFLOPs	Params (M)
YOLOv8-L	0.857	0.775	0.814	0.902	87	165.2	43.7
YOLOv5-L	0.812	0.809	0.810	0.854	91	109.6	46.5
YOLOv5-Ghost	0.773	0.772	0.773	0.853	212	11.4	5.3
LF-YOLO	0.825	0.904	0.863	0.914	178	1.8	1.1
YOLO-Xweld	0.763	0.806	0.784	0.883	164	3.6	4.6
WD-YOLO (Ours)	0.834	0.923	0.876	0.926	98	94	38.8

**Table 2:** Ablation experiment.

Method	Precision	Recall	mAP@0.5
YOLOv5-L with CBAM and BRA	0.795	0.872	0.885
WD-YOLO (no attention)	0.894	0.805	0.909
WD-YOLO with BRA	0.709	0.865	0.773
WD-YOLO with CBAM	0.901	0.772	0.875
WD-YOLO (Ours)	0.834	0.923	0.926

**Table 3:** Batch size experiment.

Method	Precision	Recall	mAP@0.5
WD-YOLO BN (bs = 2)	0.776	0.792	0.831
WD-YOLO BN (bs = 16)	0.808	0.906	0.874
WD-YOLO LN (bs = 2)	0.831	0.840	0.91
WD-YOLO LN (bs = 16)	0.834	0.923	0.926

**Table 4:** GCE module experiment.

Method	Precision	Recall	mAP@0.5
YOLOv5-L without GCE	0.187	0.334	0.216
YOLOv5-L	0.812	0.809	0.854
WD-YOLO without GCE	0.152	0.270	0.174
WD-YOLO	0.834	0.923	0.926

## Data Availability

Due to the nature of this research, participants in this study did not agree for their data to be shared publicly, so supporting data are not available.
